# Investments and Outcomes in Oral Health in Europe: Health Expenditure and the Prevalence of Dental Caries

**DOI:** 10.3390/epidemiologia7040116

**Published:** 2026-08-20

**Authors:** Cassandra Lupita, Magda-Mihaela Luca, Anca-Cristina Perpelea, Iulia Muntean, Edida Maghet, Oana Ramona Lobonț, Alexandra-Cristina Maroiu, Laura-Cristina Rusu

**Affiliations:** 1 Department of Oral Pathology, Multidisciplinary Center for Research, Evaluation, Diagnosis and Therapies in Oral Medicine, “Victor Babes” University of Medicine and Pharmacy Timisoara, Eftimie Murgu Square 2, 300041 Timisoara, Romania; 2 Department of Pediatric Dentistry, Faculty of Dental Medicine, “Victor Babes” University of Medicine and Pharmacy Timisoara, Eftimie Murgu Square 2, 300041 Timisoara, Romania; 3 Department of Organization, Professional Legislation and Management of the Dental Office, Faculty of Dental Medicine, “Carol Davila” University of Medicine and Pharmacy, 17-23 Plevnei Street, 020021 Bucharest, Romania; 4 Finance, Business Information Systems and Modeling Department, Faculty of Economics and Business Administration, West University of Timisoara, 300115 Timisoara, Romania; 5 Department of Prosthodontics, Faculty of Dental Medicine, “Victor Babes” University of Medicine and Pharmacy Timisoara, Eftimie Murgu Square 2, 300041 Timisoara, Romania

**Keywords:** oral health, total health expenditure, caries prevalence, edentulism, socioeconomic determinants, panel data analysis, Europe

## Abstract

*Background/Objectives:* Oral diseases are among the most prevalent non-communicable diseases and impose a substantial public health burden across Europe. Although general total health expenditure may reflect health-system capacity, it does not specifically measure oral healthcare financing, and evidence regarding its association with population-level oral health remains limited. The objective of this study was to assess the association between total health expenditure and oral health outcomes in European countries. The primary focus was on dental caries prevalence, while a secondary exploratory analysis examined edentulism. *Methods:* Using publicly available international databases, an ecological, unbalanced country-level panel dataset was constructed. The main caries model included 153 country-year observations from 25 European countries during 2015–2021. An extended model incorporating practicing dentist density included 109 observations from 20 countries. Two-way fixed-effects regression models with country and year effects and country-clustered standard errors were applied. Edentulism was examined using exploratory cross-sectional OLS models for 2019. *Results:* In the main model, higher total health expenditure (β = −0.00305, *p* = 0.027) and GDP per capita (β = −0.00024, *p* = 0.021) were associated with lower caries prevalence. In the extended model, only GDP per capita remained statistically significant (β = −0.00028, *p* = 0.021), whereas total health expenditure, tertiary educational attainment, and practicing dentist density were not significantly associated with caries prevalence. No statistically significant associations were observed in the exploratory edentulism analyses. *Conclusions*: Total health expenditure and GDP per capita were negatively associated with caries prevalence in the main country-level model, although the expenditure association was not maintained in the restricted extended sample. These ecological findings do not establish causality and require confirmation using more complete, oral-health-specific data.

## 1. Introduction

Oral diseases are among the most prevalent non-communicable diseases worldwide and impose a substantial cumulative burden on individuals, health systems, and societies [1,2,3]. Dental caries and tooth loss, including edentulism, are commonly used as indicators of population oral health because they reflect both current preventive conditions and long-term exposure to social, behavioral, environmental, and healthcare-related factors [2,4]. Despite improvements in oral health over recent decades, marked disparities persist between European countries and population groups, suggesting that structural and socioeconomic conditions remain relevant to the distribution of oral health outcomes [5,6].

Total health expenditure has long been recognized as a central component of health-system capacity and population health [7,8]. Greater investment may support preventive programs, healthcare infrastructure, workforce organization, and access to services [9]. A potential association with dental caries is therefore plausible at the health-system level, because prevention, early detection, and continuity of care depend partly on institutional and financial capacity.

However, total health expenditure does not specifically measure expenditure allocated to oral healthcare. Dental financing arrangements vary considerably across Europe, and dental services frequently involve greater out-of-pocket expenditure and more restricted public coverage than general medical care [9]. Consequently, any association between total health expenditure and caries prevalence should be interpreted as a contextual relationship rather than as evidence that general healthcare expenditure directly finances caries prevention.

The relationship between health expenditure and health outcomes is also influenced by socioeconomic conditions, resource-allocation efficiency, health-system organization, service coverage, and population needs [10,11]. In the field of oral health, evidence regarding the relationship between total health expenditure and oral health outcomes remains limited, partly due to inconsistent findings reported across countries and different study designs [12].

Improvements in health outcomes, including oral health, have been observed in settings characterized by higher levels of socioeconomic development, commonly represented by indicators such as gross domestic product (GDP) per capita and educational attainment [13,14]. At the population level, GDP per capita may reflect differences in material conditions, institutional resources, and healthcare capacity. Educational attainment may also capture broader social and structural characteristics related to health information, preventive environments, and patterns of healthcare utilization [15]. However, associations observed for national educational indicators cannot be interpreted as individual-level effects of education or health literacy.

Healthcare workforce availability represents another component of system capacity, with practicing dentist density frequently used as a country-level indicator of service availability [16]. Nevertheless, dentist density does not directly measure affordability, geographic distribution, effective access, service utilization, or the preventive orientation of dental care. Workforce availability may therefore contribute additional information without necessarily showing an independent association with population-level oral health outcomes [16,17].

From a methodological standpoint, cross-country analyses increasingly use panel-data methods to evaluate repeated observations while accounting for differences between countries that remain stable over time [18,19,20]. In an ecological panel, the observational units are countries rather than individuals, and repeated country-level measurements should not be interpreted as follow-up of the same individual cohort.

Fixed-effects models are particularly useful in this context because they control for time-invariant country characteristics, while year effects account for shocks and temporal patterns shared across countries [18,19,20,21]. These methods remain observational and cannot establish causal relationships or support individual-level inference.

Despite the expanding availability of international datasets on health and socioeconomic indicators, empirical evidence examining the relationship between total health expenditure and oral health outcomes across multiple European countries remains limited. Much of the existing research has concentrated on single-country studies, selected population subgroups, or specific clinical indicators rather than on population-level outcomes evaluated across comparable national contexts [14,15,17,22].

Relatively few studies have simultaneously examined health expenditure, socioeconomic development, educational attainment, and dental workforce availability within a European country-level panel framework [10,12,17,21].

The novelty of the present study lies in combining a multi-country European ecological panel with several macro-level indicators in a common analytical framework. Specifically, the study evaluates total health expenditure together with GDP per capita and tertiary educational attainment, examines the additional contribution of practicing dentist density in a restricted complete-case model, and complements the caries panel analysis with an exploratory cross-sectional assessment of edentulism.

Accordingly, the objective of this study was to investigate the association between total health expenditure and oral health outcomes across European countries, with primary emphasis on caries prevalence and a secondary exploratory analysis of edentulism. Because all variables were measured at the country level, the hypotheses concern ecological associations and do not imply individual-level mechanisms or causal effects.

The study was guided by two primary hypotheses:

- Higher total health expenditure is associated with lower country-level caries prevalence across European countries over time.
- Higher GDP per capita is associated with lower country-level caries prevalence.

Educational attainment was included as an additional socioeconomic indicator without assuming that the country-level coefficient represents an individual educational effect. Because comparable data on practicing dentist density were available for a smaller number of country-year observations, dentist density was evaluated as an additional explanatory contribution in an extended model rather than as a separate directional hypothesis.

## 2. Materials and Methods

### 2.1. Study Design and Analytical Framework

The present study followed a quantitative observational design based on secondary country-level data. An ecological panel framework was used to examine associations between total health expenditure and caries prevalence across European countries [18,20]. The unit of analysis was the country-year, and no individual participants or cohorts were followed.

Repeated annual observations were analyzed for the period 2015–2021. Because complete information was not available for every country, every year, the dataset was treated as an unbalanced panel. The primary analytical sample contained 153 unique country-year observations from 25 European countries. A restricted complete-case sample comprising 109 observations from 20 countries was used for the extended model that included practicing dentist density. The main caries sample included all country–year observations with complete data for caries prevalence, total health expenditure, GDP per capita, and tertiary educational attainment. The extended sample was constructed by additionally requiring non-missing practicing dentist-density data. No countries or years were excluded according to discretionary criteria; differences in sample composition resulted exclusively from indicator availability and the application of complete-case analysis.

The analytical framework evaluated country-level associations while accounting for unobserved characteristics that remained stable within countries and for temporal patterns shared across countries. A secondary exploratory analysis of edentulism was conducted using cross-sectional country-level data for 2019. Accordingly, the unit of analysis for the edentulism models was the country rather than the country-year.

Given the ecological and observational design, the analyses were intended to identify population-level associations rather than individual-level relationships, causal effects, or structural determinants in a mechanistic sense.

### 2.2. Data Sources and Study Population

The initial study frame comprised 25 European countries: Austria, Belgium, Bulgaria, Croatia, Cyprus, Czechia, Denmark, Estonia, Finland, France, Germany, Greece, Hungary, Ireland, Italy, Latvia, Lithuania, Luxembourg, the Netherlands, Poland, Portugal, Romania, Slovakia, Spain, and Sweden. Indicator availability differed by country and year; therefore, complete-case analytical samples were constructed for each model rather than assuming complete and balanced country coverage.

Data was obtained from publicly accessible international databases commonly used in health-services and population-health research [16,23,24,25,26]. The outcome and explanatory variables were retrieved from distinct sources and subsequently merged by country and year. Values from different databases were not combined or averaged for the same indicator. Country names and codes, reference years, measurement units, and indicator definitions were standardized before merging, and only one observation per indicator was retained for each country-year combination.

The following data sources were used:

- Caries prevalence data were obtained from the Institute for Health Metrics and Evaluation Global Burden of Disease 2023 results repository. The outcome was defined as the prevalence of caries of permanent teeth for both sexes and all ages and was reported as a proportion [23].
- Edentulism prevalence data were obtained from the World Health Organization oral-health data repository. The indicator was defined as the prevalence of edentulism among people aged 20 years or older and was reported as a percentage. The available observations referred to 2019 [24].
- Total health expenditure was obtained exclusively from the OECD Data Explorer dataset Health expenditure and financing (DSD_SHA@DF_SHA). The selected measure was expenditure (EXP_HEALTH) expressed as a percentage of GDP (PT_B1GQ) [26].
- GDP per capita data were obtained from the Eurostat dataset tec00114 and expressed as a volume index of real expenditure per capita in purchasing power standards, with EU27_2020 = 100 [25].
- Tertiary educational attainment data were obtained from Eurostat population and education statistics and expressed as the percentage of the relevant population group with tertiary education [25].
- Practising dentist density was obtained from Eurostat dataset hlth_rs_prs2. The indicator was restricted to dentists (DENT) with practising professional status (PRACT) and was expressed per 100,000 inhabitants (P_HTHAB) [25].

After data harmonization and the removal of duplicate country-year records, the main caries dataset contained 153 observations from 25 countries during 2015–2021. Because some indicators were unavailable for particular country-year combinations, the resulting panel was unbalanced. The extended caries sample, which additionally included practicing dentist density, comprised 109 observations from 20 countries.

Edentulism data were available for a single reference year and were therefore analyzed cross-sectionally. The main edentulism sample included 23 countries, while the extended sample incorporating practicing dentist density included 17 countries.

### 2.3. Statistical Analysis

The statistical analysis was carried out using the Python programming language (version 3.14). Common libraries for data analysis, such as pandas, statsmodels, and linearmodels, were used to perform the analysis. Data cleaning and transformation, and the creation of panel datasets, were achieved using custom scripts developed specifically for the study.

Caries prevalence was the dependent variable in the ecological panel analysis. Two fixed-effects regression models were estimated. The main model included total health expenditure, GDP per capita, and tertiary educational attainment as explanatory variables and was based on 153 country-year observations from 25 countries. The extended model additionally included practicing dentist density and was based on 109 observations from 20 countries.

Both caries models included country fixed effects and year fixed effects. Country fixed effects controlled for unobserved time-invariant differences between countries, while year fixed effects accounted for temporal patterns and shocks shared across countries during the study period. The inclusion of year effects partially accounted for common disruptions during the COVID-19 pandemic; however, it could not eliminate differences between countries in pandemic intensity, restrictions on dental services, or changes in access to care.

Country-clustered robust standard errors were used in both fixed-effects models to account for potential heteroskedasticity and within-country dependence. Regression coefficients were reported together with cluster-robust standard errors, two-sided *p*-values, and 95% confidence intervals.

Edentulism prevalence was analyzed cross-sectionally because comparable data were available only for 2019. The main edentulism model included total health expenditure, GDP per capita, and tertiary educational attainment and was based on 23 countries. The extended model additionally included practicing dentist density and was based on 17 countries. Both models were estimated using ordinary least squares regression with HC1 heteroskedasticity-robust standard errors. Given the single observation available for each country, country-level clustering was not applicable to the edentulism models.

Multicollinearity among the explanatory variables was assessed using the Variance Inflation Factor (VIF). All VIF values were below 1.5, indicating no evidence of problematic multicollinearity. Country-clustered standard errors were specified as a priori because the panel contained repeated observations within countries and therefore allowed for potential heteroskedasticity and within-country correlation.

Statistical significance was evaluated using a two-sided significance level of α = 0.05. All analyses were conducted using reproducible scripts, and the analytical code used for data cleaning and statistical modeling is available from the corresponding author upon reasonable request.

### 2.4. Data Availability

All datasets used in this study were obtained from publicly accessible international databases, including IHME, WHO, OECD, and Eurostat. The processed datasets and statistical code used for data cleaning and analysis are available from the corresponding author upon reasonable request. No proprietary data was used in this study.

### 2.5. Ethical Considerations

This study used aggregated country-level data obtained from publicly available databases and did not involve human participants, patient records, or identifiable personal information. Therefore, ethical approval and informed consent were not required in accordance with institutional and international research guidelines.

## 3. Results

### 3.1. Descriptive Statistics and Dataset Characteristics

After data harmonization and removal of duplicate country-year records, the main caries dataset contained 153 unique country-year observations from 25 European countries during 2015–2021. Because complete information was not available for every country in every year, the resulting dataset was an unbalanced panel. The extended caries dataset, which additionally included practicing dentist density, comprised 109 observations from 20 countries.

Edentulism prevalence was available for a single reference year and was therefore analyzed cross-sectionally. The main edentulism dataset included 23 countries, while the extended dataset incorporating practicing dentist density included 17 countries. Each country contributed a single observation to the corresponding edentulism analysis.

Table 1 presents descriptive statistics separately for the four analytical samples. In the main caries sample, mean caries prevalence was 0.374, while mean total health expenditure was 8.47% of GDP. Mean GDP per capita was 99.39 on the PPS index, and mean tertiary educational attainment was 19.17%. In the extended caries sample, the mean density of practicing dentists was 78.91 per 100,000 inhabitants. Mean edentulism prevalence was 12.54% in the main cross-sectional sample and 12.61% in the extended sample.

**Figure 1:**
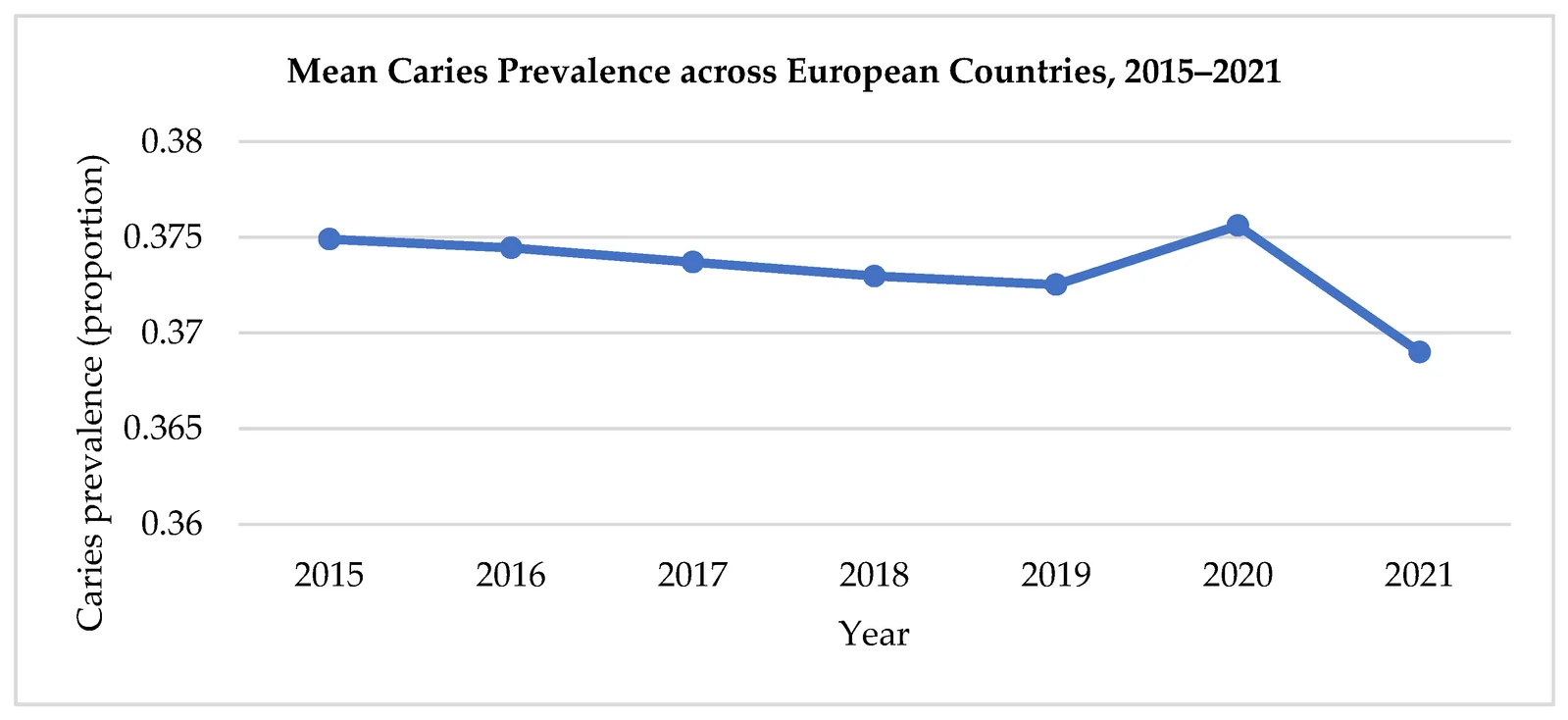
Temporal trend in caries prevalence in European countries, 2015–2021.

Mean caries prevalence remained relatively stable between 2015 and 2020, followed by a lower mean value in 2021. However, the 2021 estimate was based on only 11 countries and should not be interpreted as evidence of a general decline across all included countries (Figure 1).

### 3.2. Econometric Analysis of Caries Prevalence

Two fixed-effects regression models were estimated to examine country-level associations between total health expenditure and caries prevalence. The main model included total health expenditure, GDP per capita, and tertiary educational attainment and was based on 153 country-year observations from 25 countries. The extended model additionally included practicing dentist density and was estimated using the restricted complete-case sample of 109 observations from 20 countries. Both models included country and year fixed effects, and standard errors were clustered by country. The results are presented in Table 2.

**Figure 2:**
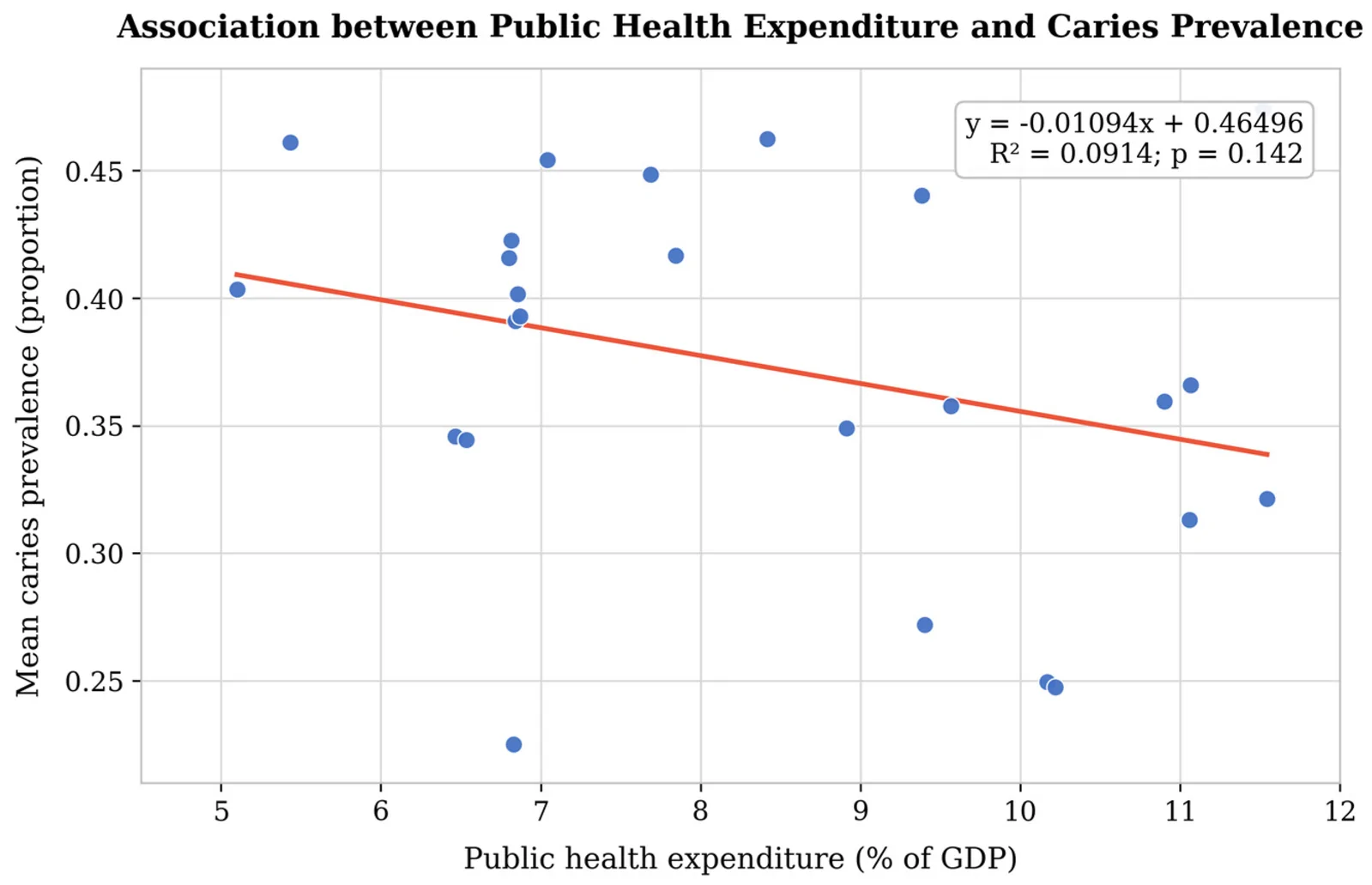
Association between mean total health expenditure and mean caries prevalence across European countries.

Each point represents one country and is based on the mean of its available country-year observations during 2015–2021. Total health expenditure is presented on the *x*-axis, and mean caries prevalence is presented on the *y*-axis. The fitted line illustrates the unadjusted cross-sectional relationship between country means and should not be interpreted as the fixed-effects regression estimate (Figure 2).

**Figure 3:**
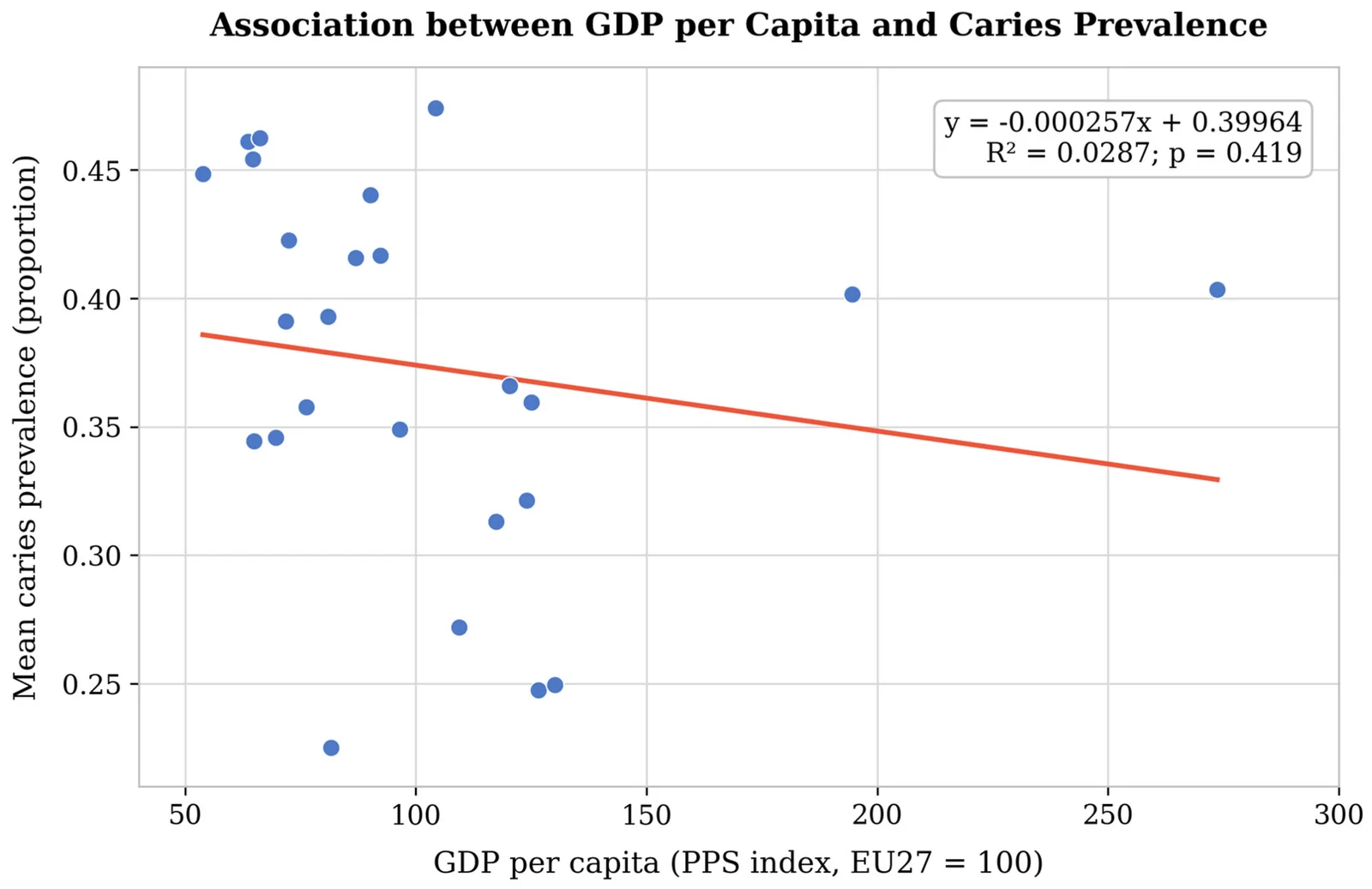
Association between mean GDP per capita and mean caries prevalence across European countries.

Each point represents one country and is based on the mean of its available country-year observations during 2015–2021. GDP per capita, expressed as a PPS index, is presented on the *x*-axis, and mean caries prevalence is presented on the *y*-axis. The fitted line illustrates the unadjusted cross-sectional relationship between country means and should not be interpreted as the fixed-effects regression estimate (Figure 3).

In the main fixed-effects model, total health expenditure was negatively associated with caries prevalence (β = −0.003054; 95% CI: −0.005731 to −0.000376; *p* = 0.027). GDP per capita also showed a statistically significant negative association (β = −0.000240; 95% CI: −0.000442 to −0.000039; *p* = 0.021). Tertiary educational attainment showed a small positive coefficient of borderline statistical significance (β = 0.000387; 95% CI: 0.000008 to 0.000766; *p* = 0.046), which was not maintained in the extended model.

In the extended model, GDP per capita remained negatively associated with caries prevalence (β = −0.000275; 95% CI: −0.000504 to −0.000047; *p* = 0.021). Total health expenditure (*p* = 0.189), tertiary educational attainment (*p* = 0.248), and practicing dentist density (*p* = 0.516) were not statistically significant.

**Figure 4:**
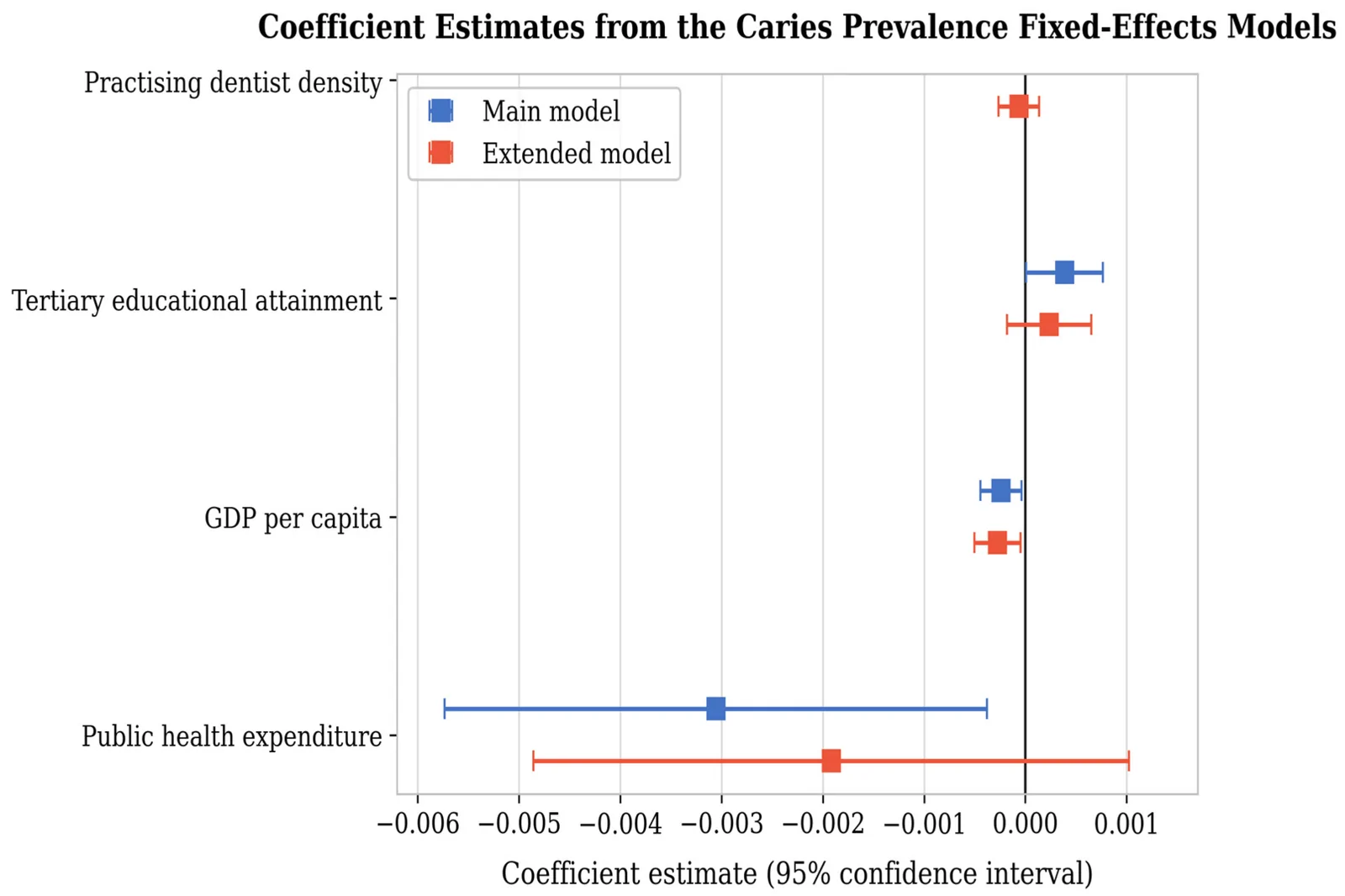
Fixed-effects coefficient estimates and 95% confidence intervals for the main and extended caries models.

Both models include country- and year-fixed effects, and confidence intervals are based on standard errors clustered by country. The main model included 153 country-year observations from 25 countries, whereas the extended model included 109 observations from 20 countries. Confidence intervals crossing zero indicate that the corresponding coefficient was not statistically significant at the 5% level (Figure 4).

The within-country R^2^ was 0.405 for the main model and 0.553 for the extended model. The explanatory variables were jointly significant in both specifications (Wald *p* = 0.014 and *p* = 0.020, respectively). These values are reported descriptively and should not be interpreted as evidence of strong predictive or causal performance.

Variance Inflation Factor values ranged from 1.04 to 1.12 in the main model and from 1.17 to 1.40 in the extended model, indicating no evidence of problematic multicollinearity. Country-clustered standard errors were used for statistical inference to account for the repeated country-level observations and potential within-country correlation.

### 3.3. Exploratory Cross-Sectional Analysis of Edentulism

Because edentulism prevalence was available for a single reference year, cross-sectional ordinary least squares regression was used to examine country-level associations with edentulism prevalence. Two exploratory models were estimated. The main model included total health expenditure, GDP per capita, and tertiary educational attainment and was based on 23 countries. The extended model additionally included practicing dentist density and was based on 17 countries. HC1 heteroskedasticity-robust standard errors were used in both models. The results are presented in Table 3.

No statistically significant associations were identified in either edentulism model. In the main model, total health expenditure, GDP per capita, and tertiary educational attainment were not significantly associated with edentulism prevalence. The model explained a limited proportion of the observed cross-sectional variation (R^2^ = 0.028; adjusted R^2^ = −0.125).

In the extended model, practicing dentist density was also not statistically significant (β = −0.0591; 95% CI: −0.1432 to 0.0251; *p* = 0.152). Although the extended model showed a higher R^2^ value (R^2^ = 0.357; adjusted R^2^ = 0.143), this estimate should be interpreted cautiously because the model was based on only 17 countries.

The absence of statistically significant associations should not be interpreted as evidence that structural or health-system conditions are unrelated to edentulism. Edentulism is a cumulative life-course outcome, while the present analysis was based on a single cross-sectional observation for each country and had limited statistical power.

## 4. Discussion

### 4.1. Main Findings

The major result of this ecological analysis is that increased public health spending and GDP per capita have been linked to the decreased prevalence of dental caries in the primary fixed-effects model. In the extended model, which was based on a smaller and differently composed complete-case sample, only GDP per capita remained statistically significant, whereas total health expenditure, tertiary educational attainment, and practicing dentist density were not significantly associated with caries prevalence.

There were no associations with statistically significant differences found in the secondary cross-sectional models of edentulism. In the case of edentulism being considered a lifetime outcome, this finding should be interpreted cautiously owing to the fact that the analysis has used only one data point for each country.

### 4.2. Interpretation and Comparison with Previous Studies

The negative association between total health expenditure and caries prevalence identified in the main model is consistent with the literature linking health-system resources and socioeconomic conditions to population health outcomes [9,12]. However, total health expenditure is not a direct measure of expenditure on oral healthcare. Dental-service financing differs substantially across European countries and frequently includes considerable out-of-pocket expenditure. The indicator should therefore be interpreted as a broad measure of health-system expenditure rather than as a measure of investment in oral prevention or treatment.

The association between total health expenditure and caries prevalence was not statistically significant in the extended model, which used a smaller and differently composed complete-case sample. Consequently, the evidence for an independent association between total health expenditure and caries prevalence remains suggestive rather than conclusive. The loss of statistical significance cannot be attributed solely to the inclusion of practicing dentist density because the analytical sample also changed.

GDP per capita had a negative effect on caries prevalence in both models. This result is supported by previous findings concerning the social patterning of oral diseases and their relation to socioeconomic factors [13,27]. In general, higher economic development of countries could be associated with better healthcare infrastructure, living conditions, and preventive opportunities. These explanations were not considered in this study and could not be discussed in terms of causality.

While tertiary education showed a weak positive correlation with caries prevalence in the main regression model, it did not have statistical significance in the extended regression model. This contrasts with the typically favorable effect that education had on oral health outcomes at the individual level [15]. In view of the current study employing aggregated country-level data, the coefficient may be due to differential national reporting, country-specific demographics, access to diagnosis, and other country-level variables that were not accounted for. It does not mean that having more education increases caries prevalence.

The density of practicing dentists was not statistically significant in the extended regression model. According to previous studies in the field of health systems, the number of providers alone might not be sufficient to represent cost, distribution, service delivery, and prevention orientation [21,28]. Moreover, the regression analysis on dentist density was performed using a smaller number of countries than other models. Thus, it does not suggest that the oral health workforce is not important, only that there is no significant independent correlation of dentists’ density with caries prevalence.

No statistically significant associations were found in the exploratory edentulism models. Edentulism reflects cumulative exposures, treatment histories, and healthcare access over an extended period, whereas the analysis was restricted to a single reference year. The cross-sectional design and small number of countries may, therefore, have limited the ability to identify associations between current structural indicators and an outcome that developed over the life course.

### 4.3. Public Health and Policy Implications

The findings indicate that oral health outcomes should be investigated in relation to their broader socioeconomic and health-system context. The results suggest that health-financing indicators may be relevant to future research on differences in oral health across European countries, alongside economic development, care, service organization, and oral-health-specific expenditure. However, the present ecological associations do not demonstrate that changing total health expenditure would necessarily alter caries prevalence.

The absence of a statistically significant association between practicing dentist density and caries prevalence should not be interpreted as evidence that the oral-health workforce is unimportant. Provider density does not capture geographic distribution, affordability, service utilization, insurance coverage, or the balance between preventive and restorative care. Future policy evaluations should therefore consider not only workforce numbers but also the accessibility, financing, distribution, and preventive orientation of dental services.

### 4.4. Strengths and Limitations

A strength of this study is the integration of oral-health, socioeconomic, health-expenditure, and workforce indicators from established international databases within a comparative European framework. The use of two-way fixed-effects models allowed the analysis to account for time-invariant differences between countries and common year-specific changes. Country-clustered standard errors were used to address heteroskedasticity and within-country dependence, while the main and extended models made it possible to distinguish the primary analysis from the smaller complete-case analysis that included practicing dentist density.

Several limitations should nevertheless be considered. First, this was an ecological observational study based on aggregated country-level data. The reported associations cannot be interpreted at the individual level, and causal relationships cannot be established. Reverse causality is also possible: countries with a greater oral-disease burden may modify healthcare expenditure in response to population needs. The potential direction of the relationship between expenditure and oral health therefore cannot be determined from the present analysis.

Second, total health expenditure does not specifically measure expenditure on oral healthcare, preventive dental programs, insurance coverage, or out-of-pocket payments. Dental-care financing and access arrangements differ substantially across European countries, and these differences were not directly captured. Furthermore, combining information from IHME, WHO, OECD, and Eurostat may introduce heterogeneity related to indicator definitions, estimation procedures, reporting practices, and population coverage, despite efforts to harmonize the datasets.

Third, the study period included the COVID-19 pandemic. Although year fixed effects were included to account for common temporal shocks, the pandemic may have affected countries differently in terms of healthcare expenditure, dental-service accessibility, data reporting, and population behavior. Country-specific pandemic effects may therefore remain as residual confounding.

Fourth, relevant behavioral and contextual variables, including sugar consumption, smoking, oral-hygiene practices, use of preventive services, fluoride exposure, dental insurance coverage, and out-of-pocket expenditure—were not consistently available and could not be included. The models may consequently be affected by residual confounding. Moreover, contemporaneous expenditure was analyzed, although changes in health-system financing may influence oral-health outcomes only after a time lag.

Finally, the caries dataset was unbalanced, and the extended model included fewer observations and countries because of missing dentist-density data. Differences between the main and extended estimates may therefore reflect changes in sample composition. The exploratory edentulism analysis was further limited by its cross-sectional design, the small number of countries, and the cumulative life-course nature of edentulism. These limitations substantially restrict the ability to identify associations between current structural indicators and tooth loss.

### 4.5. Future Research Directions

Future research should consider employing longer time series at the country level to explore delayed relationships between health care financing and oral health indicators. The use of lagged expenditures, natural experiments, or an appropriate application of instrumental variables could assist in understanding the issue of temporal sequencing and possible endogeneity of the problem under consideration. Detailed metrics on expenditures related to oral health care, prevention services, availability of dental insurance, out-of-pocket costs, utilization, fluoride consumption, diet, and other behavioral risks need to be used. In the cases where the data is available, subnational and individual-level analysis could supplement the results at the country level, prevent the ecological fallacy, and detect any existing inequalities that cannot be detected from the aggregate data.

## 5. Conclusions

GDP per capita showed a consistent negative association with caries prevalence in both country-level fixed-effects models. Total health expenditure was negatively associated with caries prevalence only in the main model, and this association was not maintained in the smaller extended complete-case sample. The evidence concerning total health expenditure should therefore be considered suggestive and specification dependent. No statistically significant associations were identified in the exploratory cross-sectional analyses of edentulism. These findings represent ecological associations and do not establish causal relationships.

These findings require confirmation using longer and more detailed time-series data, including oral-health-specific expenditure, service provision and utilization, out-of-pocket payments, behavioral risk factors, and potential time-lag effects. Individual-level and subnational data would also be valuable for examining mechanisms and inequalities that cannot be assessed using aggregated national data.

## Figures and Tables

**Table 1:** Descriptive statistics of study variables across European countries.

Sample/Variable	N	Mean	Standard Deviation	Minimum	Maximum
Main caries sample					
Caries prevalence (proportion)	153	0.374	0.071	0.222	0.476
Total health expenditure (% GDP)	153	8.47	1.95	4.90	12.70
GDP per capita (PPS index)	153	99.39	40.32	49.00	279.00
Tertiary educational attainment (%)	153	19.17	7.73	4.50	36.80
Extended caries sample					
Caries prevalence (proportion)	109	0.364	0.074	0.223	0.476
Total health expenditure (% GDP)	109	8.45	2.09	4.90	12.50
GDP per capita (PPS index)	109	98.69	38.73	49.00	279.00
Tertiary educational attainment (%)	109	17.83	7.26	4.50	36.80
Practicing dentist density (per 100,000)	109	78.91	17.00	33.18	113.50
Main edentulism sample					
Edentulism prevalence (%)	23	12.54	1.83	8.86	16.85
Total health expenditure (% GDP)	23	8.44	1.84	5.70	11.40
GDP per capita (PPS index)	23	96.43	30.32	55.00	190.00
Tertiary educational attainment (%)	23	20.05	8.00	7.60	35.10
Extended edentulism sample					
Edentulism prevalence (%)	17	12.61	1.86	9.60	16.85
Total health expenditure (% GDP)	17	8.51	2.04	5.70	11.40
GDP per capita (PPS index)	17	95.12	24.10	55.00	128.00
Tertiary educational attainment (%)	17	18.94	7.19	7.60	35.10
Practicing dentist density (per 100,000)	17	81.83	16.77	56.51	113.50

Note: N represents unique country-year observations in the caries samples and countries in the cross-sectional edentulism samples. The main models include total health expenditure, GDP per capita, and tertiary educational attainment. The extended models additionally include practicing dentist density.

**Table 2:** Two-way fixed-effects models examining country-level associations with caries prevalence.

Model/Variable	β	Cluster-Robust SE	95% CI	*p*-Value
Main model: 153 observations, 25 countries				
Total health expenditure (% GDP)	−0.003054	0.001297	[−0.005731, −0.000376]	0.027
GDP per capita (PPS index)	−0.000240	0.000098	[−0.000442, −0.000039]	0.021
Tertiary educational attainment (%)	0.000387	0.000184	[0.000008, 0.000766]	0.046
Extended model: 109 observations, 20 countries				
Total health expenditure (% GDP)	−0.001915	0.001405	[−0.004855, 0.001025]	0.189
GDP per capita (PPS index)	−0.000275	0.000109	[−0.000504, −0.000047]	0.021
Tertiary educational attainment (%)	0.000237	0.000199	[−0.000179, 0.000653]	0.248
Practicing dentist density (per 100,000)	−0.000064	0.000097	[−0.000266, 0.000138]	0.516

Notes: Both models include country- and year-fixed effects. Standard errors are clustered by country. The main model includes total health expenditure, GDP per capita, and tertiary educational attainment. The extended model additionally includes practicing dentist density. CI, confidence interval.

**Table 3:** Exploratory cross-sectional OLS models examining country-level associations with edentulism prevalence in 2019.

Model/Variable	β	HC1 Robust SE	95% CI	*p*-Value
Main model: 23 countries				
Total health expenditure (% GDP)	0.0534	0.1618	[−0.2853, 0.3921]	0.745
GDP per capita (PPS index)	0.0053	0.0088	[−0.0132, 0.0238]	0.557
Tertiary educational attainment (%)	0.0235	0.0578	[−0.0976, 0.1445]	0.689
Extended model: 17 countries				
Total health expenditure (% GDP)	−0.2457	0.5290	[−1.3983, 0.9068]	0.651
GDP per capita (PPS index)	0.0077	0.0561	[−0.1146, 0.1300]	0.894
Tertiary educational attainment (%)	0.0722	0.0688	[−0.0777, 0.2221]	0.315
Practicing dentist density (per 100,000)	−0.0591	0.0386	[−0.1432, 0.0251]	0.152

Notes: HC1 heteroskedasticity-robust standard errors were used. The main model included total health expenditure, GDP per capita, and tertiary educational attainment. The extended model additionally included practicing dentist density. CI, confidence interval.

## Data Availability

The datasets analyzed in this study were derived from publicly available international databases, including the Global Burden of Disease (GBD), World Health Organization (WHO), Eurostat, and Organization for Economic Cooperation and Development (OECD) repositories. Processed datasets and analytical code used to generate the reported results are available from the corresponding author upon reasonable request.

## Abbreviations

The following abbreviations are used in this manuscript:

GDP	Gross Domestic Product
VIF	Variance Inflation Factor
OECD	Organization for Economic Cooperation and Development
OLS	Ordinary Least Squares
CI	Confidence Interval
PPS	Purchasing Power Standard
IHME	Institute for Health Metrics and Evaluation
HC1	Heteroskedasticity-Consistent Type 1
COVID-19	Coronavirus Disease 2019
GBD	Global Burden of Disease
WHO	World Health Organization
